# Impact of transforming growth factor beta 1 on normal and thyroid cancer side population cells

**DOI:** 10.1007/s12020-022-02990-4

**Published:** 2022-02-03

**Authors:** Nani Md Latar, Kamilla Mahkamova, Joanna Elson, Isha Karnik, Rachel Sutherland, Sebastian Aspinall, Annette Meeson

**Affiliations:** 1 Department of Surgery, Faculty of Medicine, Universiti Kebangsaan Malaysia Medical Centre, Kuala Lumpur, Malaysia; 2 Newcastle University Bioscience Institute, Newcastle University, International Centre for Life, Central Parkway, Newcastle upon Tyne, NE1 3BZ UK; 3 Department of General Surgery, Aberdeen Royal Infirmary, Foresterhill, Aberdeen, AB252ZN UK

**Keywords:** Thyroid, Thyroid cancer, Side population cells, TGF-β

## Abstract

**Purpose:**

To determine the impact of exogenous transforming growth factor beta 1 (TGF-β1) on side population (SP) cells isolated from normal, papillary thyroid cancer and anaplastic thyroid cancer cell lines and from human thyroid tissues.

**Methods:**

All cell populations were stained with Hoechst 33342 and analysed using dual wavelength flow cytometry to identify SP cells. This SP assay was used to assess the impact of TGF-β1 treatment and withdrawal of treatment on SP percentages. Semi-quantitative and quantitative PCR were used for molecular analysis of cells pre and post TGF-β1 treatment.

**Results:**

All cell lines expressed mRNA for both *TGFB1* and its receptors, as well as showing variable expression of *CDH1* and *CDH2*, with expressing of *CDH1* being highest and *CDH2* being lowest in the normal cell line. Exposure to exogenous TGF-β1 resulted in a reduction in mRNA expression of *ABCG2* compared to controls which was significant between control and treated cancer cell lines. SP cells were isolated from primary human thyroid tissues, with numbers being significantly higher in papillary thyroid cancers. Exposure to TGF-β1 decreased the SP percentage in both thyroid cancer cell lines and completely abrogated these cells in the primary papillary thyroid cancer cultures. On withdrawal of TGF-β1 the SP phenotype was restored in the cancer cell lines and SP percentages increased to above that of untreated cells.

**Conclusions:**

TGF-β1 exposure transiently regulates thyroid cancer SP cells, leading to a reduction in SP percentages, while withdrawal of TGF-β1 results in restoration of the SP phenotype.

## Introduction

Transforming growth factor beta (TGFβ) is a pleiotropic cytokine that under normal conditions is involved in a range of cellular process including but not limited to regulating cell growth, proliferation and differentiation. The role of TGF-β in cancer progression is still a matter of debate, as it has been described as having both tumour suppression and tumour promoting potential, depending on stage of cancer progression [1]. Moreover, TGF-β signalling and/or hypoxia have also been reported to drive epithelial to mesenchymal transition (EMT) in many cancers contributing to cancer progression (reviewed in refs. [2, 3]), including thyroid cancers were EMT makers have been found to be overexpressed in more aggressive and metastatic thyroid tumours in vivo and in vitro [4–8]. In the thyroid, TGF-β1 has also been reported to increase the expression of HMGA1 at both the gene and protein level in the thyroid cancer cell line SW579. While HMGA1 has been reported to be involved in the progression of several types of cancers including thyroid, this study supports a role for TGF-β1 in its induction [9].

SP cells have been shown to be present in a number of different cancers and cancer cell lines and have been demonstrated to be more tumorigenic and drug resistant than non-SP cells (NSPs) [10]. SP cell behaviour has been reported to be regulated at some levels when exposed to endogenous TGF-β1 in several cancers including breast cancer, ovarian cancer and pancreatic cancer [11–13]. For example in the breast cancer cell line MCF7 SP it was shown that MCF7 SP abundance could be depleted by TGF- β1 directed EMT and that this was associated with a decrease in *ABCG2* expression. Moreover, removal of TGF-β1 resulted in restoration of *ABCG2* expression and SP cell numbers [11]. While SP cells of the pancreatic cancer cell lines PANC-1 and Capan-2 were more responsive than NSPs (bulk cells without the SP fraction) to TGF-β1 switching their phenotypic traits for epithelial to mesenchymal and back upon reversal of TGF-β1 treatment [13].

We have previously reported on the presence of SP cells in the normal thyroid cell line N-thy ori-3-1, papillary thyroid cancer (PTC) cell line BCPAP and the ATC cell line SW1736 and reported that these cancer SP cells are responsive to hypoxia, with an increase in cell number upon hypoxia exposure [14]. In addition, we reported on functional differences in terms of migration and invasion potential in the SW1736 SP above that of the N-thy ori-3-1 SP [15].

The objectives of this study were to determine the impact on thyroid SP cells exposed to endogenous TGF-β1 and then to determine the impact of removal of endogenous TGF-β1 on these cells. We hypothesized that fluctuations in TGF-β1 levels would show a transient effect on SP cell behaviour and might go some way in explaining why targeting of the TGF-β pathway alone has had little impact in the clinical setting for successfully treating a number of different cancers [16].

## Materials and methods

### Cell lines

Cell lines used in this study included the PTC cell line BCPAP and the ATC cell line SW1736, gifts from G. Brabant (Universitats Klinikum, Germany) and C. McCabe (University of Birmingham, UK), while the N-thy ori-3-1 was purchased (ECACC, UK). All cell lines were authenticated prior to use using STR fingerprinting.

### Culturing of cells lines

Cell culture was carried out as described previously [15]. But in brief cells were cultured in appropriate base media, RPMI 1640 for the N-thy ori-3-1 and BCPAP and DMEM GlutaMAX for the SW1736 (both from Gibco, UK). All media was supplemented with 10% FBS L-glutamine and penicillin/streptomycin. Once 80% confluent cells were harvested and used for analysis.

### Ethics and tissue collection

This study was performed according to the amended Declaration of Helsinki and informed consent from patients. As part of patient consent, all patients consented for results of this study to be used for scientific publication. Tissue used in this study was surplus to diagnostic requirements and collected under ethical approval REC number 13/NE/0026 2013. Tissue was made available for research purposes on the day of surgery.

### Isolation of RNA from tissue

In brief, 1 ml of Tri reagent (Sigma, UK) was added to 50–100 mg of tissue, this was then dissociated using a MACS dissociator (MIlentyi Biotec, UK) programme RNA-01, the aqueous layer was then removed and centrifuged 11,000 *g* for 10 min. 200 μl of chloroform was then added to this and left at room temp for 3 min. This was then centrifuged as above for 15 min and clear aqueous layer containing RNA collected for further processing using the RNeasy MinElute Kit (Qiagen, UK) as per manufactures instructions.

### Semi-quantitative PCR analysis

RNA from tissue and from cells was used for cDNA synthesised using the Tetro-cDNA synthesis kit (Bioline, UK), as per manufacturer’s instructions. PCR was performed as describe previously [15]. Primers used in this study included *ABCB1* F:5′-CTGACGTCATCGCTGGTTTC-3′, R:5′- ATTTC CTGCTGTCTGCATTGTGA-3′; *ABCG2* F:5′-GAGCGCACGCATCCTGAGAT-3′, R:5′ TCATTGGAAGCTGTCGCGGG-3′; and *GAPDH* F: 5'-GCCTTCTCCATGGTGGTGGTGAA-3', R: 5'-GCACCGTCAAGGCTGAGAAC-3', which was used as a loading control.

### Isolation and culture of primary tissue-derived cells

Tissue was minced into @2 mm pieces manually, this was then digested in collagenase IV 20 mg/ml (Gibco, UK) and DNase 0.01% (Worthington, UK) in 20 ml of complete Roswell Park Memorial Institute 1640 (RPMI-1640) media in a shaking water bath at 37 °C for 90 min. This was the filtered using a 70 µM cells strainer, pelleted and the resulting pellet treated with lysis buffer (23 ml of 0.15 m NH_4_Cl pH 7.5) to remove red blood cells. The cells were then re-pelleted, lysis buffer discarded and the cells, isolated from all tissue types, were cultured in Humanised seven homoeostatic additives thyroid media (h7H media—see Table 1 for additives), under standard tissue culture conditions until 80% confluent, then used for further analysis. All dissociation steps in primary tissue-derived cells were performed using the Tryple/Glucose (1 g/L) combination.

**Table 1:** Humanised seven media additives

Additive	Final concentration per litre	
Somatostatin	50 ng/l	
Thyroid-stimulating hormone (TSH)	40 mIU/l	
Insulin	25 mIU/l	
Cortisol	23 nM/l	
Recombinant human growth hormone protein	0.2 μg/l	
Apotransferrin	5 mg/l	
Sodium iodide	10 μg/l	
Sodium selenite	75 μg/l	
Zinc	863 μg/l	
Iron	834 μg/l	
Sodium L-ascorbate	15 mg/l	
L-glutathione reduced	0.2 mg/l	
DL ± -α-tocopherol	0.5 mg/l	
DL-α-tocopherol acetate		0.5 mg/l
Sodium pyruvate		220 mg/l
Glucose		1800 mg/L
Ethanol (final concentration contributed from diluents of the above reagents)		0.0177%

### Real-time PCR

Real time was performed as described previously [17]. Taqman primers/probes (Applied Biosystems, UK) were used to examine gene expression, with primer/probes being specific for: *TGFB1* (Hs_00998133_m1), *TGFBR1* (Hs_00610320_m1), *TGFBR2* (Hs_00234253_m1), *CDH1* (Hs_0.023894_m1), *CDH2* (Hs_00983056_m1), *ABCG2* (Hs_01053790_m1) and *GAPDH* (Hs_02758994_g1). The comparative ΔΔCT method was used to assess relative mRNA expression by normalising to *GAPDH* and expressing values as fold change relative to N-thy ori-3-1 or to untreated cells. Real-time PCR was performed using the Quant Studio™7 Flex Real-Time PCR Machine (Life Technologies).

### SP assay

For tissue-derived SP cells, h7H was removed from cultures and cells were washed three times with phosphate buffered saline (PBS) before being detached from the flask using Tryple Express (Gibco, UK). Resulting cell suspension were stained, 1 × 10^6^ cells per ml of RPMI-1640 media, containing 1 µg/ml Hoechst 33342 dye. Cells were incubated for 60 min at 37 °C. Assay was then completed, data analysed, and SP phenotype confirmed by the addition of Verapamil as described previously [18]. Assay and data analysis for cell line SP was performed as described previously [15]. Conditions to obtain the highest SP percentage have been optimised for each cell line used in this study. The N-Thy ori-3-1, BCPAP and SW1736 needed 7, 3 and 5 μg/mL Hoechst 33342 respectively. The incubation time for N-thy ori-3-1 and BCPAP was similar at 60 min, while the SW1736 was incubated for 90 min. Verapamil was used to inhibit dye efflux, with all cells being incubated with 100 μM/mL of verapamil for 15 min before the final wash and analysis.

Gating strategy was performed using the forward (related to cell size) and side scatter (related to cell granularity) dot plots to exclude debris as described [18]. Next, dead cells were exclude based on propidium iodine (PI) dye uptake. This was done by adding PI at 2 μg/ml to all samples prior to FACs analysis as described previously [15]. PI is unable to pass through intact cell membranes and therefore only stains cells that have compromised membranes allowing for gating out of dead cells based on their PI positivity, leaving only inclusion of viable PI negative cells for analysis/sorting. This was followed by gating for single cells using the width to height ratio on Hoechst labelling plotted on Hoechst blue (3-355/405/50-A) versus Hoechst red (3-355/405/50-H) axis. This gating strategy was designed to analyse only live, single cells.

### TGF-β1 treatment and reversal of treatment of cell lines cells and tissue-derived cells

Cells for all cell lines were plated into 100 mm dishes (Corning) and 24 h after plating, treated with pre-optimised concentrations of TGF-β1 [R&D Systems, Inc., Abingdon, UK] 10 ng/mL TGF-β1 for the N-thy ori-3-1 and 1 ng/ml for BCPAP and SW1736 and incubated for 72 h. Following this, SP analysis was performed as described previously [15].

To study the impact of TGF-β1 reversal on the SP percentage, media containing exogenous TGF-β1 was removed. Cells were then washed with PBS and new pre-warmed media added before being incubated for another 72 h, the cells were then assayed for the presence of SP. In every step, following treatment and reversal of treatment of TGF-β1, untreated cells were used as control samples.

For tissue-derived cells, at 80% confluency, plated cells were treated with 1 ng/ml TGF-β1 for 14 days and then assayed for SP percentage as described previously [15]. Controls were cells cultured for 14 days but not exposed to exogenous TGF-β1.

To confirm that any changes in SP cell percentage following treatment with TGF-β1, were specifically a result of involvement of TGF-β1, cells of the SW1736 cell line were treated with 3 mM SB-505124 (Sigma, UK).

### Statistical analysis

One-way ANOVA and Tukey post-hoc test was used to determine the significance of the difference seen in SP % obtained from the normal, benign and malignant thyroid tissue-derived cells. *P*-value of <0.05 was considered significant. In this study *R*^2^ is a measure of explanatory power in this logistic regression model were *R*^2^ is used to estimate whether the regression model can be reliably used or not to explain the relationship between the studied variables. Therefore, this was used to explain the relationship between the percentage of SP in relation to gender, age and nodule size. The regression model was run in a stratified manner looking at the influence of each factor on SP percentage (see Table 2).

**Table 2:** Details of patients age, gender, source of tissue, size of tissue and percentage SP isolated from tissue

Tissues		Patient	SP (%)	Gender		Age		Size of tissue (mm)
Source of normal tissue								
From completion thyroidectomy		1	0.1	Female		50		n/a
With follicular adenoma		2	0.2	Female		42		n/a
From completion thyroidectomy		3	0	Female		35		n/a
With MNG		4	0.2	Female		78		n/a
With follicular adenoma		5	0.1	Female		56		n/a
With follicular adenoma		6	0	Female		37		n/a
With follicular adenoma		7	0.1	Female		45		n/a
With MNG		8	0.1	Female		34		n/a
From completion thyroidectomy		9	0.4	Female		32		n/a
With follicular adenoma		10	0.1	Male		47		n/a
From completion thyroidectomy		11	0.1	Male		48		n/a
Source of benign tissue								
Colloid nodule		1	0.2	Female		30		26
Follicular adenoma		2	0.2	Female		42		20
Graves’ disease		3	0.7	Female		30		n/a
MNG		4	0.2	Female		78		30
Follicular adenoma		5	0.1	Male		47		20
Follicular adenoma		6	0.1	Female		45		20
Follicular adenoma		7	0.1	Female		37		18
Follicular adenoma		8	0.1	Female		35		13
Source of malignant tissue								
Papillary cancer		1	1.6	Male		48		75
Papillary cancer		2	0.8	Male		64		13
Papillary cancer		3	0.6	Female		56		30
Source of tissue for semi-quantitative PCR		Patient	n/a	Gender		Age		n/a
Normal	1		n/a	Female		27		n/a
Papillary cancer	2		n/a	Female		17		n/a
Follicular adenoma	3		n/a	Female		37		n/a
Follicular cancer	4		n/a	Female		23		n/a

*MNG* multinodular goitre, *n/a* not available

## Results

### Bulk cells of the N-thy ori-3-1, BCPAP and SW1736 cell lines express TGF-β receptors and TGF-β1 and show variable expression of EMT markers

**Fig. 1:**
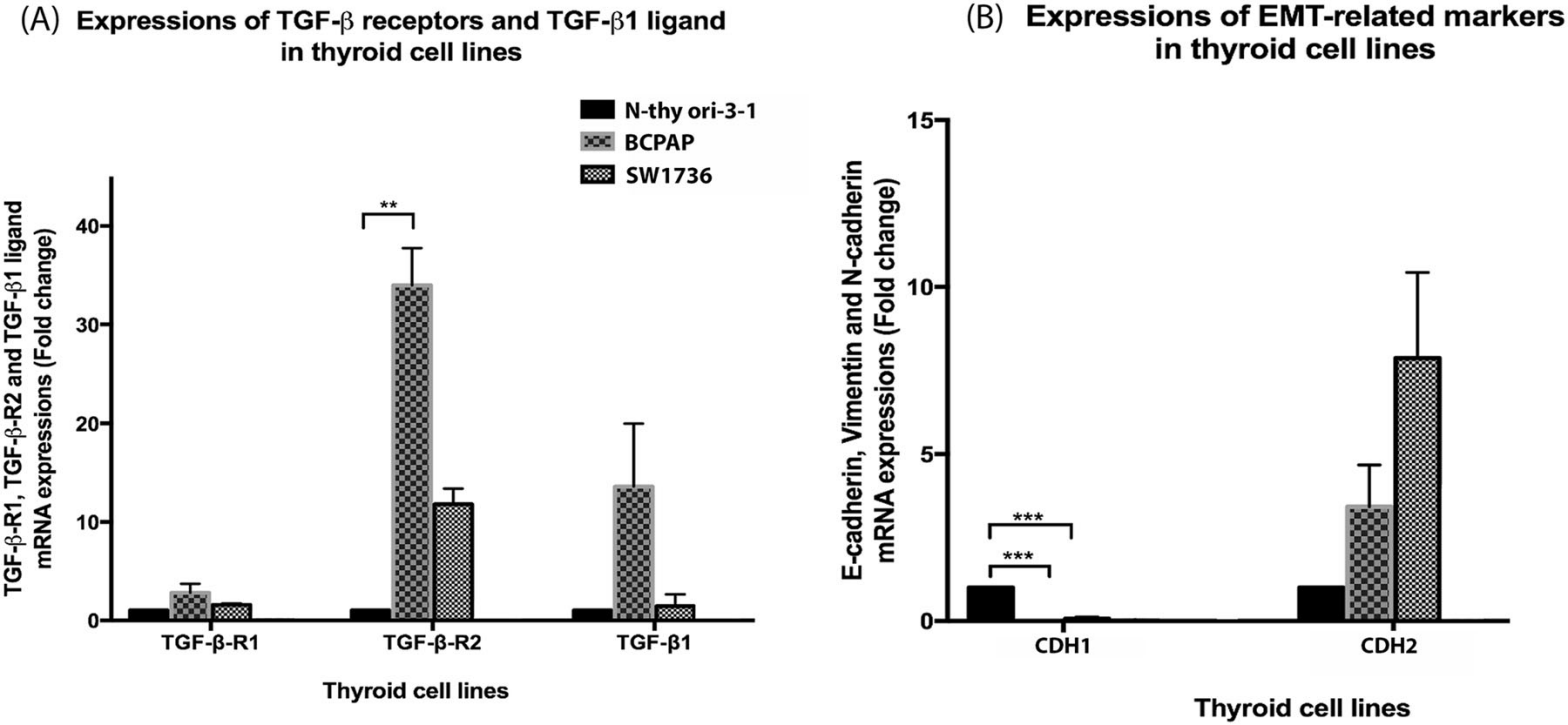
Cells of the N-thy ori-3-1, BCPAP and SW1736 cell lines express TGFB receptors, TGFB1, CDH1 and CDH2. mRNA expressions of *TGFBR1, TGFBR2 and TGFB1* were determined using real-time PCR from the above-mentioned cell lines (**a**). Note the significant difference in expression detected between the BCPAP and N thy ori 3-1 in expression levels for *TGFBR2* **p < 0.05. Expression of mRNA fold change for *CDH1* and *CDH2* were also determined for each cell line (**b**). Note significantly higher fold expression for *CDH1* mRNA by the N thy ori 3-1 cell above that for both cancer cell lines ***p < 0.001. All 3 cell lines expressed *CDH2*. In all cases, data was normalised to *GAPDH* and presented as fold change from the N-Thy ori-3-1 cells. Each bar represents the mean ± SEM from three cell passages

Real-time PCR was used to determine expression of mRNA level for *TGFBR1, TGFBR2, TGFB1*, *CDH1* and *CDH2* in the N-thy ori-3-1, BCPAP and SW1736 cell lines. All 3 cell lines showed expression of *TGFB1* and both receptors (Fig. 1A). However, only *TGFBR2* was expressed at significantly higher levels in the BCPAP cell line above that of expression in the N-thy ori-3-1 cell line (*p* < 0.005). Although not significant, there was also higher expression of this receptor in the SW1736 cell line above that on the N-thy ori-3-1 cell line. BCPAP also showed higher expression levels of *TGFB1* than the N-thy ori-3-1 cells, but this was not significant. There was little difference in expression levels of *TGFBR1* between the 3 cell lines.

*CDH1* was expressed at significantly higher levels in the N-thy ori-3-1 cells than in the BCPAP or SW1736 cell lines (*p* < 0.001). All three cell lines expressed CDH2 with expression being lowest in the N-thy ori-3-1 compared with both cancer cell lines and with the highest level of expression being detected in the SW1736 cell line, but these differences in expression were not significant (Fig. 1B).

### *ABCG2* expression is reduced in bulk N-thy ori-3-1, BCPAP and SW1736 cells after exposure to exogenous TGF-β1

**Fig. 2:**
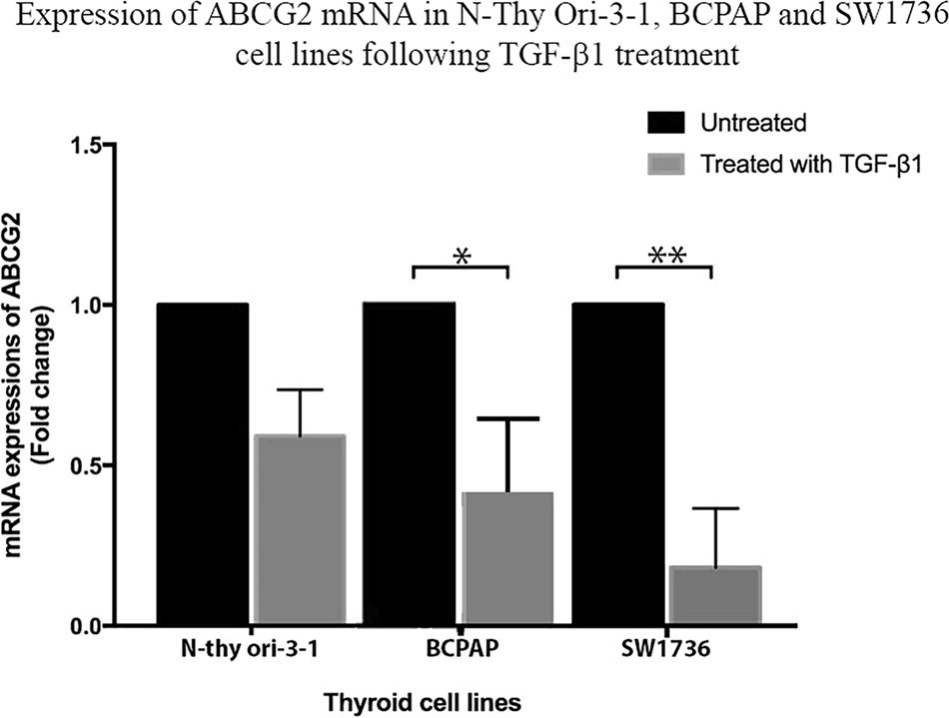
mRNA expression of *ABCG2* following TGF-β1 treatment of N thy ori 3-1, BCPAP and SW1736 cells is reduced. Fold change in mRNA expression of *ABCG2* was determined in untreated and TGF-β1 treated cells for all 3 cell lines using real-time PCR. Note that post-treatment there was a non-significant reduction in *ABCG2* expression in N thy ori-3-1 cells. While for treated BCPAP and SW1736 there was a significant reduction in *ABCG2* expression levels being *p < 0.05 and **p < 0.005 respectively. In all cases, data are presented as mean fold change ± SEM and *N* = 3. Untreated cells were used as normal control

Bulk N-thy ori-3-1, BCPAP and SW1736 cells were treated for 72 h with TGF-β1. Following treatment cells were harvested and the level of *ABCG2* mRNA expression was compared to controls (untreated cells) for all lines. In the N-thy ori-3-1 cells there was a non-significant reduction in expression of *ABCG2* whereas, for both cancer lines expression was significantly reduced when comparing BCPAP to control (*p* < 0.05) and SW1736 to control (*p* < 0.005) (Fig. 2).

### SP cell percentage in the BCPAP and SW1736 cell line were reduced after exposure to endogenous TGF-β1

**Fig. 3:**
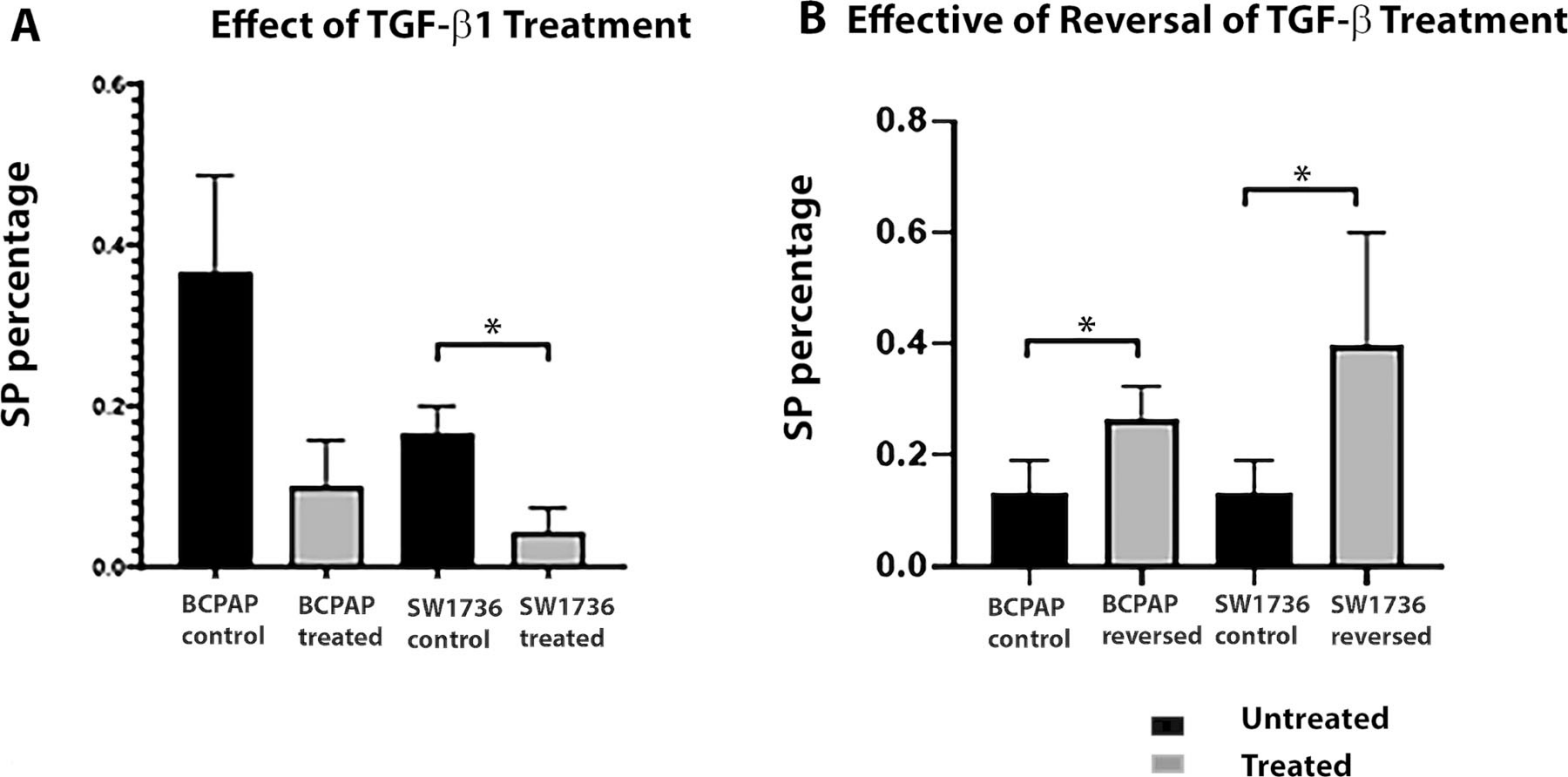
TGF-β1 treatment of the BCPAP and SW1736 resulted in a reduction In SP percentage which was restored on removal of TGF-β1. (**a**) Bar chart representing SP percentage of BCPAP and SW1736 cell cultures treated with TGF-β1 1 ng/ml for 72 h. A reduction in SP percentage in both cancer cell lines was observed following treatment but this was only significantly different for treated SW1736 cells compared to control *p < 0.05. (**b**) Bar chart representing SP cell percentage 72 h are removal of TGF-β1 treatment in both cancer cell lines. Note after removal of treatment SP cell percentages increased significantly for both cell lines compared to controls *p < 0.05. Data presented as mean ± SD, and for 3 biological replicates, *N* = 3

We also determined the impact of exposure of the cancer cell lines to TGF-β1 on the percentage of SP cells. For both cancer cell lines there was a reduction in SP percentage however, this was only significant for the SW1736 cells when comparing control (untreated SW1736) cells to treated SW1736 (*p* < 0.05) (Fig. 3A). To determine that the reduction/loss of detectable SP cells following exposure to TGF-β1 was due specifically to involvement of the TGF-β pathway, cells of the SW1736 cell line were also treated with 3 mM SB505124 (a TGF-β1 receptor inhibitor) alone or with a combination of TGF-β1 and inhibitor, and then analysed using the SP assay. We observed that under both conditions the SP percentage was restored to or even increased above that of SP % of untreated cells, while TGF-β1 treatment alone resulted in a loss of the SP (Supplementary Fig. 1).

### SP cell percentages increase following withdrawal of TGF-β1 in the BCPAP and SW1736 cell lines

Next, we examined the impact of withdrawal of TGF-β1. Cells were harvested at 72 h after removal of TGF-β1 and assayed for SP. The SP population of both cancer cells lines was restored and increased, this increase was significant when comparing control and experimental SP cells for both BCPAP and SW1736 to their respective controls (*p* < 0.05) (Fig. 3B).

### SP cells can be isolated from normal, benign diseased and malignant thyroid tissue

**Fig. 4:**
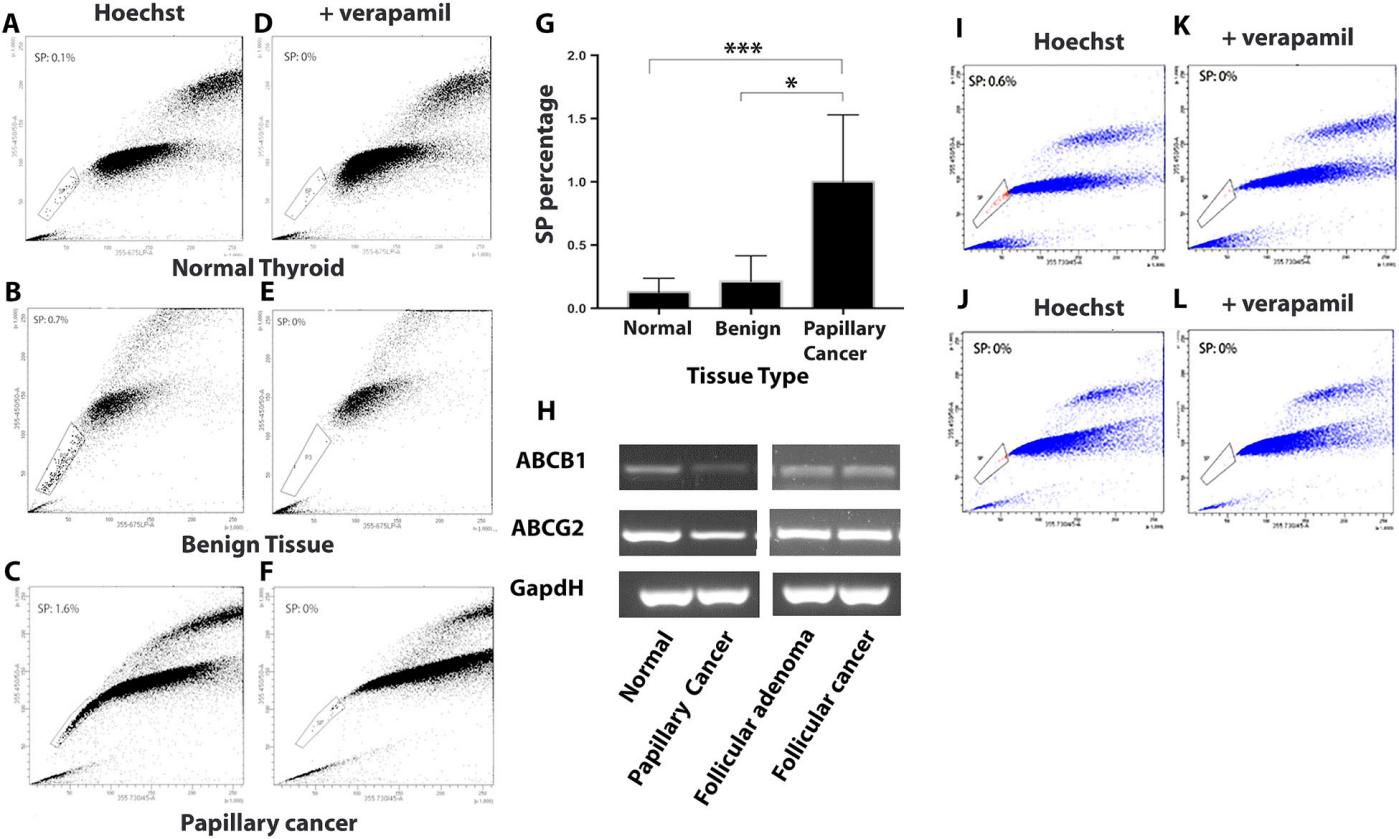
Thyroid tissues contain SP cells and express the ABC transporters ABCB1 and ABCG2, SP percentages are highest in PTC, SP cells isolated from PTC are reduced when treatment with TGF-β1. Representative SP FACS profiles are shown for SP cells from NT (**a**), from BT (**b**) and from PTC (**c**). In all cases representative SP profiles are shown for each sample with the inclusion of verapamil to confirm the SP phenotype (**d**, **e**, **f**) respectively. A bar chart representing the mean SP percentage for each tissue type is shown (**g**) the percentages are only significant when comparing PTC SP to normal SP ***p < 0.001 and PTC SP to BT SP *p < 0.05. Results were based on analysis of 11 NT, 8 BT and 3 PTC patient derived cultures. Semi-quantitative PCR results showing expression of mRNA for *ABCB1* and *ABCG2* are shown for several different thyroid tissue types including normal, benign diseased and tumour tissue (**h**). *GAPDH* was used as a loading control. Representative SP FACS profile of PTC tissue-derived cultures treated with TGF-β1. (**i**) A representative FACS profile of an SP assay for an untreated PTC culture which has an SP of 0.6%. while following treatment this percentage of SP was reduced to below detectable levels (**j**). Representative FACs profiles for SP assay of these cultures but with the inclusion of verapamil are also shown (**k**, **l**). *N* = 3 different patient samples were used for this analysis

SP cells were isolated from primary cultures derived from normal thyroid tissue (NT), benign diseased thyroid tissue (BT) and PTC. In the case of the SP from NT, SP could only be detected in 9 out of 11 samples with SP percentage ranging from 0 to 0.4%, with the mean being 0.12%. A representative FACS plot showing an NT SP cell population of 0.1% is shown (Fig. 4A). For SP isolated from BT, 8 samples were analysed, and all contained SP, SP percentage ranging from 0.1 to 0.7%, mean = 0.213%. A representative FACS profile of cultured cells isolated from BT showing an SP cell population of 0.7% is shown (Fig. 4B). For SP isolated from PTC, 3 samples were examined with SP percentage ranging from 0.6 to 1.6%, mean = 1.0%. A representative FACS profile of cultured cells isolated from PTC showing an SP population of 1.6% is shown (Fig. 4C). The SP phenotype for all 3 tissue-derived SP populations was confirmed by the addition of 100 µM verapamil, representative profiles of this for NT, BT and PTC derived cells are also shown (Fig. 4D–F). A bar graph representing mean SP cell population % from 11 NT, 8 BT and 3 PTC cultures (Fig. 4G) shows that there is a significant difference in SP cell population % between PTC and NT SP (*p* < 0.001) and between PTC and BT SP (*p* < 0.05), with the % being higher in both cases for SP cells isolated from PTC.

We also examined 4 different thyroid tissue samples for mRNA expression of *ABCB1* and *ABCG2* and all expressed both transporters, although expression was higher in all cases for *ABCG2* (Fig. 4H) SP data for each individual tissue analysed and patient information is provided (Table 2).

### TGF-β1 treatment of PTC tissue-derived cells results in a loss of the SP cell population

Cells derived from both NT and PTC were treated with TGF-β1 and then assayed for the presence of SP cells, controls were untreated cells. In all cases cells were cultured for 14 days before being assayed for the presence of SP. In the case of cells from NT (*n* = 3 patients) when untreated there was a small increase in SP percentage for *n* = 2 but no change for the third donor (FACS data not shown). For cells derived from PTC (*n* = 3 patients, see Table 2) following treatment we could detect in all cases a decrease in PTC SP cell percentage (mean SP in untreated cells was 0.4% while in the treated mean SP was 0%). Note a representative FACS plot showing in control (untreated) primary PTC cultures an SP of 0.6% was detected (Fig. 4I) but after 14 days of TGF-β1 treatment, note the percentage of PTC SP is now undetectable (Fig. 4J). In all cases controls included the addition of Verapamil to confirm the SP phenotype. Data representing the SP percentages for all 3 NT and 3 PTC cultures for both TGF-β1 treated and untreated cells, and verapamil controls for each culture, are provided (Supplementary Fig. 2).

### Statistical analysis comparing SP cell percentages to patient demographics

No statistical relationships were observed between SP percentages and patient age, gender or size of nodule/tumour (see Table 2 for details of tissue samples used) using logistic regression modelling and *R*^2^.

## Discussion

In the thyroid, SP cells have been shown to be present in normal and nodular goitres and in normal and thyroid cancer cell lines [19, 20]. In the present study, we have shown that we can isolate SP cells from primary cell cultures derived from NT, BT and PTC. Our SP analysis revealed the SP percentage to be significantly higher in PTC compared to NT and BT. Our statistical analysis revealed no correlation between SP cell number, patient age, gender or size of nodule/tumour, but this data should be treated cautiously as patient numbers were small. The presence of SP cells from thyroid tissue has previously been identified in primary human thyroid cell cultures established from human goitres at passage 2 where SP was 0.1% or from growth factor stimulated thyrospheres which are enriched for SP (increased to 5%) [19]. In this study we may also have expanded SP cell numbers by culturing primary cells from all 3 tissue types in h7H media until 80% confluent.

ABC transporters, particularly ABCG2 and ABCB1, have been linked to the SP phenotype for SP isolated from several other tissue types [21–24] and cancer cell lines [17, 21, 25]. *ABCG2* expression has been reported to be more highly expressed in a number of thyroid cancer cell lines above that of their NSP [20] and *ABCB1* has been reported to be expressed in the N-thy ori-3-1 and BCPAP cell lines [15]. In addition, higher ABCG2 expression has also been reported in the solid component of PTC (and while still controversial PTC containing solid components have been suggested to be linked to poorer prognostic outcomes) [26]. Therefore, we also examined several thyroid tissues for expression of these genes and showed that regardless of tissue origin (normal, benign or cancerous) we could detect mRNA for both transporters.

Based on our previous published study showing we could isolate SP cells from the N-thy ori-3-1, BCPAP and SW1736 cell lines, that have characteristics of stem cells [15], we employed these cell lines in this study. However, as with use of all cell lines, it is important to note that overtime their characteristics may change. For example, BCPAP was originally classified as a PTC cell line [27]. However, during the in vitro evolution of this cell line, its DNA synthesis/replication mechanisms have been partially lost and it now more closely resembles a poorly differentiated thyroid cancer.

Therefore, this needs to be taken into consideration when interpreting results generated using this cell line [28, 29]. We examined bulk cells for the expression of markers associated with TGF-β and EMT and observed expression of both *TGFβ* ligand and receptors in all 3 cell lines. Our analysis showed that only in the case of *TGFBR2* was there significantly higher expression of this receptor in the BCPAP cell line above that of expression in the N thy ori-3-1. It has been reported in a study comparing *TGFB1* mRNA levels in both benign nodules and PTC that the levels of *TGFB1* expression were significantly higher in the PTC [24]. While in an earlier study examining expression of *TGFB1* in both PTC and normal thyroid it was also shown that *TGFB1* was overexpressed in the PTC tissue [8].

In terms of EMT marker expression, *CDH1* was expressed at higher levels in the N-thy ori-3-1 cell line whereas *CDH2* was more highly expressed in the cancer cell lines. These results are perhaps not unexpected as down regulation of *CDH1* and increased expression of *CDH2* is pivotal for the onset of EMT (reviewed in ref. [30]).

Having determined that the thyroid cell line models we were employing expressed markers that predicted TGF-β responsiveness we then wanted to determine if TGF-β1 treatment impacted on *ABCG2* expression. We observed that following treatment mRNA levels of *ABCG2* were reduced significantly when comparing both cancer cell lines to control. We then went onto determine if this treatment impacted on SP cell percentage, as would be suggested by the reduction in *ABCG2* expression and were able to determine that for both the cancer cell lines there was a reduction in SP percentage, but this was only significant when comparing control and treated SW1736. To determine if this was a reversible loss of the SP phenotype, we then removed the exogenous TGFβ and observed restoration of the SP phenotype and an increase in SP abundance in both cancer cell lines compared to controls. To confirm that these changes were due specifically to the impact of TGF-β1, we treated cells of the SW1736 cell line with a TGF-β1 receptor inhibitor and demonstrated that in the presence of this inhibitor the SW1736 SP was restored. This is in line with a study of MCF7 breast cancer SP cells which also showed a reduction in *ABCG2* expression and a reduction in SP numbers following TGF-β treatment, both of which were reversable on removal of exogenous TGF-β1 [11].

We then examined the impact of TGFβ treatment on SP cells of primary PTC and NT and observed that while treatment had little effect on SP of NT (see supplementary Fig. 2), the percentage of SP cells in primary PTC cultures were reduced to below detectable levels using our SP protocol and gating strategy.

While therapeutic targeting of TGF-β as a means of treating cancers has as yet not lived up to the results observed in some pre-clinical studies, we suggest limitations might be due in part to cell type being targeted and EMT being partial. For example, in ovarian cancer, higher numbers of SP cells have been reported to be present in ovarian cancer cell lines. When treated with TGF-β1 these SP cell numbers are reduced and driven towards a more mesenchymal cell phenotype. They display an increase in expression of some EMT markers. For example, snail1 an important regulator of EMT.

Silencing of snail1 then resulted in the ovarian cancer SP cells showing a change in mRNA expression increasing expression of epithelial markers and decreasing expression of mesenchymal markers, again suggesting that regulation of the EMT process can impact on SP cells’ ability to undergo mesenchymal transformation [12].

Our data supports a transient role for TGF-β in regulating thyroid cancer SP cell behaviour and further points to the need for a better understanding of the complexed role of TGF-β in cancer, including in regulation of cancer stem cells.

## Supplementary information

Supplementary Figure 1

Supplementary Figure 2

Supplementary Figure Legends
