# Supplementary figures and images for: Impact of transforming growth factor beta 1 on normal and thyroid cancer side population cells
Source: Endocrine. 2022 Feb 3;76(2):359–68. doi: 10.1007/s12020-022-02990-4 (PMC9068642; doi:10.1007/s12020-022-02990-4)

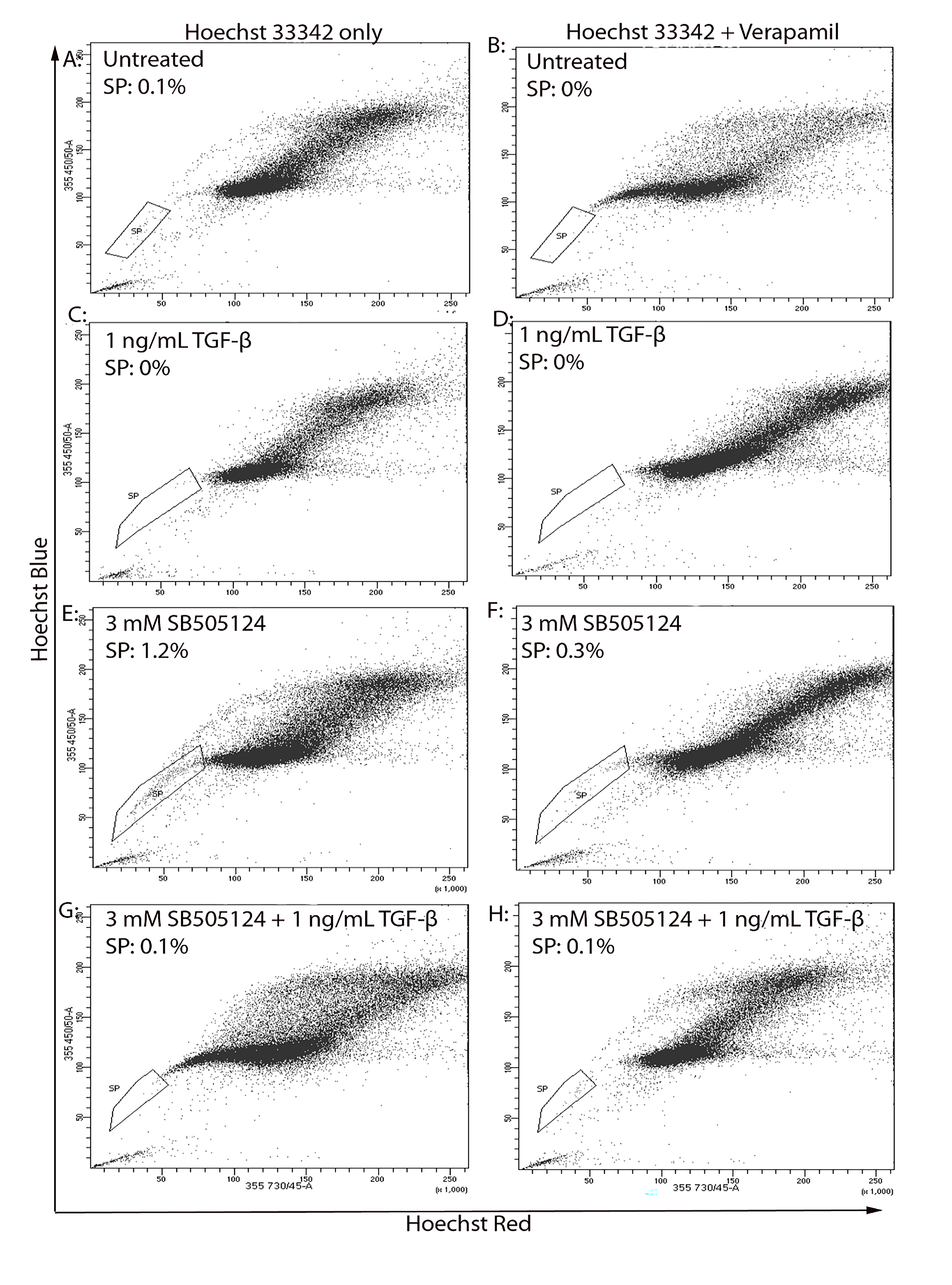

Supplement: Supplementary file 1 — Supplementary Figure 1 [file 12020_2022_2990_MOESM1_ESM.tif]
