# Supplementary material for: Impact of transforming growth factor beta 1 on normal and thyroid cancer side population cells
Source: Endocrine. 2022 Feb 3;76(2):359–68. doi: 10.1007/s12020-022-02990-4 (PMC9068642; doi:10.1007/s12020-022-02990-4)
Supplement: Supplementary file 3 — Supplementary Figure Legends [file 12020_2022_2990_MOESM3_ESM.docx]

**Supplementary Figure 1. Addition of the TGF-B1 receptor inhibitor SB505124 to SW1736 cells restores the SP.** Representative FACS images show in (a) An SP of 0.1 % is present in untreated SP of the SW1736 cell line. (c) When SW1736 cells are treated with 1 ng/ml TGF-β1 no SP can be detected. (e) When SW1736 cells are treated with 3mM of the inhibitor only an SP of 1.2 % is detected and (g) when cells are treated with both TGF-β1 and inhibitor the SP percentage is restored to that of untreated SP. (b, d, f and h) are cells treated as for (a, c, e and g) respectively but with the addition of Verapamil (VP).

**Supplementary Figure 2. Impact of TGF-β1 treatment on tissue derived SP leads to loss of PTC SP whereas for NT SP there is a small increase in SP**. SP percentages are represented as a bar chart. Data is representative of 3 NT donors and 3 PTC donors all treated as single biological samples. Note the data represents 3 NT cultures and 3 PTC cultures, and two experimental groups those not exposed to TGF-β1 = untreated and those exposed to TGF-β1 = Treated. In all cases a proportion of the cells for each donor culture treated and untreated with TGF-β1 were also treated with VP.
